# Humics-Functionalized Iron(III) Oxyhydroxides as Promising Nanoferrotherapeutics: Synthesis, Characterization, and Efficacy in Iron Delivery

**DOI:** 10.3390/nano15181400

**Published:** 2025-09-11

**Authors:** Anastasiya M. Zhirkova, Maria V. Zykova, Evgeny E. Buyko, Karina A. Ushakova, Vladimir V. Ivanov, Denis A. Pankratov, Elena V. Udut, Lyudmila A. Azarkina, Sergey R. Bashirov, Evgenii V. Plotnikov, Alexey N. Pestryakov, Mikhail V. Belousov, Irina V. Perminova

**Affiliations:** 1 Department of Chemistry, Lomonosov Moscow State University, Leninskiye Gory 1-3, Moscow 119991, Russia; kiorika@bk.ru (K.A.U.); pankratov@radio.chem.msu.ru (D.A.P.); iperminova@gmail.com (I.V.P.); 2 Pharmaceutical Faculty, Siberian State Medical University, Tomsk 634050, Russia; buykoevgen@yandex.ru (E.E.B.); ivanovvv1953@gmail.com (V.V.I.); evu8@mail.ru (E.V.U.); ludmila_logvinova@mail.ru (L.A.A.); bars-tomsk@rambler.ru (S.R.B.); mvb63@mail.ru (M.V.B.); 3 Research School of Chemistry and Applied Biomedical Sciences, Tomsk Polytechnic University, Tomsk 634050, Russia; plotnikov.e@mail.ru (E.V.P.); pestryakov@tpu.ru (A.N.P.)

**Keywords:** humic substances, iron nanoparticles, synthesis, alcohol precipitation, cell culture, cytotoxicity, bioavailability

## Abstract

Iron deficiency anemia (IDA) remains a global health challenge. This study pioneers the use of humic substances (HS) as natural, biocompatible macroligands to develop safer and more effective nanoferrotherapeutics. We synthesized a series of nanoscale Fe(III) oxyhydroxide complexes stabilized by different HS, employing various solvents (ethanol, isopropanol, and acetone) and precipitation methods to isolate fractions with optimized properties. The nanocomposites were comprehensively characterized using inductively coupled plasma atomic emission spectrometry, total organic carbon analysis, X-ray diffraction, transmission electron microscopy, and Mössbauer spectroscopy. Cytotoxicity and iron bioavailability of all HS-Fe(III) formulations were assessed in Caco-2 intestinal epithelial cells. The type of HS and precipitation conditions significantly influenced the nanocomposites’ properties, yielding spherical nanoparticles (1–2 nm) of ferrihydrite or goethite. Physicochemical analysis confirmed that solvent-driven fractionation effectively tailored the nanocomposites’ size, crystallinity, and elemental composition. All HS-Fe(III) formulations demonstrated exceptional cytocompatibility, starkly contrasting the significant cytotoxicity of the reference drug Ferrum Lek^®^. Several complexes, particularly CHSFe-Et67, surpassed Ferrum Lek^®^ in cellular iron uptake efficiency. We conclude that HS are a highly promising platform for developing effective and safe iron-delivery nanoferrotherapeutics, leveraging their natural polyfunctionality to enhance bioavailability and mitigate toxicity.

## 1. Introduction

Iron deficiency anemia (IDA), characterized by an insufficient level of functional iron, primarily within hemoglobin, represents a major global health burden, which affects nearly one-tenth of the world’s population according to WHO [1,2]. Its clinical significance is also provided by the prevalence of vulnerable groups, such as children, women of reproductive age, pregnant women, the elderly, and so on [2,3,4,5].

The established therapeutic strategy for treatment of iron deficiency (sideropenia) mandates prolonged oral iron supplementation for at least six months after hemoglobin levels normalize to fully replenish iron stores [1,2]. Clinicians choose from over 30 monocomponent or combination formulations based on Fe(II) (mainly sulfate salts) or Fe(III) (notably polynuclear iron(III)-hydroxide polymaltose complex, IPC) [1,6]. However, both classes of iron drugs face significant limitations. Fe(II) salts, which are absorbed passively, frequently cause gastrointestinal oxidative stress (in >20% of patients) and other adverse effects such as epigastric pain, metallic taste, nausea, vomiting, diarrhea, and constipation, consequences of rapid surges in serum Fe^2+^ [7,8]. Fe(III)-IPC complexes are consumed by active transport and mitigate free radical generation and food/drug interactions through redox inertness [7]. Still, their bioavailability is compromised by hydrolysis and precipitation. Furthermore, IPC therapy carries a risk of allergic reactions, including anaphylaxis [8]. Consequently, there is a critical need for novel, safer ligands to deliver Fe(III) effectively for IDA management.

Humic substances (HS) present a promising solution [9,10,11,12,13]. Humic substances are natural macroligands. They are biorefractory, polydisperse, supramolecular and are characterized by a high amount of diverse functional groups (e.g., carboxyl, phenolic, quinone, amide, ester) [9]. The polyfunctionality coupled to substantial surface area and reactivity (it encompasses ion exchange, redox processes, and van der Waals forces) underpins their biological interactions. Humic substances exhibit inherent biocompatibility and low toxicity across a wide concentration range, modulating cellular and systemic metabolism [10,11,12]. Crucially, HS function as potent polydentate complexing agent. A demonstrated correlation exists between a metal’s toxicity and its binding affinity for HS [13], highlighting their role not only in detoxification but also as potential carriers for essential metals like iron. Some of the studies have proven their efficacy in stabilizing iron oxyhydroxide nanoparticles in water, thus promoting colloidal stability, particle size, and conserving bioactive forms of Fe^3+^ [14,15,16].

Previously, our research group demonstrated the benefits of using HS for the synthesis and stabilization of iron (III) oxyhydroxides in nanoscale forms and showed that they are bioavailable to plants [17,18,19,20,21]. However, the iron compounds obtained were poorly soluble and not suitable for correcting human iron deficiency.

Lipophilicity is an important property, which determines solubility and bioavailability of pharmaceutical compounds. Lipophilic compounds have an increased ability to penetrate the lipid bilayer of cell membranes including the intestinal epithelium. However, excessive lipophilicity can be a problem as well: such molecules might be insoluble in aqueous solutions. An optimal lipophilic-hydrophilic balance of the molecule is the main condition for its effective transport through intestinal epithelium and for the subsequent intake of iron into the bloodstream [22].

One of the methods of fractionation of complex mixtures by lipophilicity is precipitation with a use of organic solvents [23,24]. A change in polarity of the medium via addition of solvent (e.g., alcohol) allows for precipitation of fractions with different lipophilicity [25]. The complexation capabilities, nanoscale stabilization, and the ability to vary lipophilicity, combined with the absence of specific enzymatic pathways for HS catabolism in humans, provides an effective mechanism for achieving sustained iron release.

Current investigation pioneers the development of humic macroligand-stabilized nanoscale iron(III) oxyhydroxide complexes. This study aimed to synthesize HS-functionalized iron(III)-based nanostructures, comprehensively characterize their physicochemical properties (structure, size, and morphology), and assess cytotoxicity and in vitro iron bioavailability for future application as advanced nanoferrotherapeutics against IDA. Special attention is given to a use of alcohol precipitation for isolation of fractions with high bioavailability.

## 2. Materials and Methods

### 2.1. Materials

Anhydrous FeCl_3_ (chemically pure grade, UD-Bio, Shenzhen, China) was used as a source of iron in the syntheses. Sodium bicarbonate NaHCO_3_ (analytically pure grade) was used for the hydrolysis of iron salts. Commercial preparations of humic substances were used as ligands: sodium fulvate isolated from peat (FA) FulvagRa 90% (Humintech Ltd., Grevenbroich, Germany), potassium humate (CHP) isolated from leonardite (Powhumus 85%, Humintech Ltd., Grevenbroich, Germany), and sodium humate isolated from Sakhalin coаl (CHS) (Sakhalin humates LLC, Sakhalin, Russia). Sodium hydroxide (NaOH, analytical grade) and hydrochloric acid (HCl, reagent grade) were used for pH adjustment. For comparative purposes, the pharmaceutical Ferrum Lek (50 mg/mL, Lek d.d., Ljubljana, Slovenia) was procured as an injectable solution. All solutions were prepared using highly purified water (18.2 MΩ·cm) obtained from a Millipore Simplicity 185 system.

### 2.2. Synthesis Iron(III) Oxyhydroxide Nanoparticles Stabilized with Humic Substances

The synthesis of humic-stabilized iron(III) oxyhydroxide was performed in three stages: (i) preparation of freshly precipitated iron(III) hydroxide, (ii) its interaction with humic macroligands, and (iii) isolation of the target product by alcohol precipitation.

#### 2.2.1. Preparation of Freshly Precipitated Iron(III) Hydroxide

For the synthesis of iron(III) hydroxide, 4.87 g (0.03 mol) of anhydrous FeCl_3_ was dissolved in 125 mL of distilled water. The resulting solution was centrifuged at 6000 rpm for 5 min and the insoluble precipitate was removed. The supernatant was cooled in an ice bath to a temperature of 7–10 °C. In parallel, 7.9 g (0.09 mol) NaHCO_3_ was dissolved in 125 mL of distilled water. NaHCO_3_ solution was slowly added dropwiseto the FeCl_3_ solution with constant stirring. The mixing was continued for 10 min until a dark brown precipitate formed. Then the ice bath was removed and mixing continued for another 60 min at pH = 7, the value of which was maintained by adding an additional amount of NaHCO_3_ solution as needed. The resulting precipitate is Fe(OH)_3_ was separated by centrifugation (5 min, 6000 rpm), washed with distilled water, resuspended by vortex mixing, and centrifuged again to remove residual salts.

#### 2.2.2. Production of Iron(III) Oxyhydroxide Nanoparticles Stabilized with Humic Substances

Fulvic acids (FA, FulvagRa, 90%) or humic acids (CHP, Powhumus, 85%; CHS, Sakhalin humates) were used as humic macroligands. A humic substance solution of humic substances was prepared by dissolving 12.4 g of HS in 120 mL of distilled water, yielding a concentration of 10.3% *w*/*v*. The resulting solution was centrifuged at 12,000 rpm for 5 min to remove insoluble impurities, after which it was heated to 65–70 °C. A total of 8 mL of 10 M NaOH was successively added dropwise to the preheated solution with constant stirring, followed by freshly precipitated Fe(OH)_3_. The mixture was stirred at a temperature of 65–70 °C for 40 min. As a result of the reaction, a precipitate of iron(III) hydroxide nanoparticles stabilized by humic macroligands was formed. At the end of the synthesis, the suspension was cooled to room temperature and acidified with 1 M HCl to pH = 6.5. The volume of the suspension was adjusted to 300 mL, with the final concentration of HS 41.3 mg/mL.

#### 2.2.3. Isolation of Fe(III)-HS Samples by Alcohol Precipitation

Two variants of precipitation were carried out: parallel (I) and sequential (II).

(I) Parallel precipitation: The final Fe(III)-HS suspension was divided into several solutions. Varying volumes of solvent (ethanol, acetone, or isopropanol) were added to each aliquot to induce precipitation. The suspensions were then centrifuged at 7000 rpm for 5 min and supernatant was decanted. A total of 5 mL of filler liquid was added to the resulting precipitate, resuspending and then centrifuged again, washing the precipitate of salts.

(II) Sequential Precipitation: A predetermined volume of solvent was added to the working mixture. The resulting suspension was centrifuged, and the separated precipitate was washed twice to remove salts. The supernatant was retained and a subsequent portion of solvent was added to it, thereby gradually changing the polarity of the medium. At each stage of precipitation, the fraction was separated and washed according to the protocoldescribed above. Consequently, a series of fractions differing in polarity and composition was obtained.

Ethanol, acetone, and isopropanol were used as organic solvents for precipitation. An example of the conditions for parallel and sequential precipitation of Fe(III)-HS samples is shown in Table 1. During parallel precipitation, the volume of the working mixture was 50 mL, and a fixed amount of alcohol corresponding to the desired concentration was added to each mixture. In the sequential scheme, the initial volume of the solution was 100 mL, and the solvent was added in stages of 25 or 50 mL. Table 1 shows the amount of added solvent required to achieve a given alcohol concentration in the system (for example, 25 mL of alcohol was added to go from 20% to 33%, and 50 mL was added from 60% to 67%).

All isolated precipitates were dried in a vacuum oven at 45 °C for 24 h.

### 2.3. Characterization of Iron(III) Oxyhydroxide Nanoparticles Stabilized with Humic Substances

Quantitative determination of iron, sodium, and potassium concentrations in aqueous samples was carried out using inductively coupled plasma atomic emission spectrometry (ICP-AES) on an Agilent 5100 instrument (Agilent Technologies, Santa Clara, CA, USA). Dissolved and total organic carbon (DOC and TOC) contents were determined using a TOC-L CSN analyzer (Shimadzu, Kyoto, Japan). Calibration was performed using standard solutions: potassium hydrogen phthalate (for organic carbon) and a sodium carbonate/bicarbonate mixture (for inorganic carbon).

Solubility was determined by dispersing 150 mg of the sample in 1 mL of distilled water under magnetic stirring for 30 min. The resulting suspension was transferred to 2 mL centrifuge tubes and centrifuged at 7000 rpm for 5 min. After separation of the supernatant, the mass of the insoluble residue was measured. Solubility was calculated as the difference between the initial sample mass and the mass of the residue, and expressed in milligrams of dissolved substance per milliliter of solution.

X-ray diffraction (XRD) analysis was carried out using a Rigaku D/MAX2000 diffractometer equipped with a rotating copper anode and a graphite monochromator, operating in Bragg–Brentano geometry. Radiation from the CuKα_1,2_ line was used. The resulting diffraction patterns were identified by comparison with entries in the PDF-2 database.

Transmission electron microscopy (TEM) was performed using a Zeiss Libra 200MC transmission electron microscope operating in bright-field mode at an accelerating voltage of 200 kV. Electron diffraction patterns were obtained from selected areas with a diameter of 2 μm.

Mössbauer spectroscopy was performed at temperatures of 296 ± 3 K and 77.7 ± 0.3 K using a ^57^Co source in a rhodium matrix. The signal-to-noise ratio of the recorded spectra did not exceed 2%. Spectra were processed at high resolution (1024 channels) using SpectRelax 3.4 software. Isomer shifts are reported relative to metallic α-Fe. Low-temperature spectra were obtained using a closed-loop CFPR-221-MESS cryostat designed for Mössbauer spectroscopy without liquid cryogens.

### 2.4. Cytotoxicity Assessment

Cytotoxicity of humic-stabilized iron complexes and the reference compound Ferrum Lek^®^ (Ferrum Lek, Ljubljana, Slovenia) towards Caco-2 intestinal epithelial cells was evaluated using the Neutral Red Uptake assay [26] to determine safe concentration ranges for subsequent iron uptake studies. The Caco-2 cell line represents a physiologically relevant and extensively utilized in vitro model for investigating intestinal iron transport, accumulation, and bioavailability, making it highly appropriate for this study [27,28,29]. Cells were exposed to concentrations ranging from 3.125 to 800 μg/mL. After incubation, viability was assessed spectrophotometrically at 540 nm (reference 650 nm) using a Tecan Infinite 200 PRO M Plex plate reader (Tecan Group Ltd., Männedorf, Switzerland).

### 2.5. Cellular Iron Uptake

Intracellular iron levels following exposure to the Fe(III)-HS complexes and Ferrum Lek^®^ were quantified using the ferrozine-based colorimetric assay. This method exploits ferrozine’s high affinity and specificity for iron, enabling robust and reliable quantification of total cellular iron content after a reduction step, as established in prior research [30,31]. Caco-2 cells were seeded at 100,000 cells/well in 12-well plates in 1 mL complete DMEM/F-12 medium (Gibco, Thermo Fisher Scientific Inc., Waltham, MA, USA) and allowed to adhere overnight. Cells were then treated with the test samples or Ferrum Lek^®^ to a final iron(III) concentration of 50 μg/mL for 24 h. Following treatment, intracellular iron content was quantified using a modified ferrozine-based assay [30]. Briefly, cell lysates underwent acid digestion with concentrated HCl (1 h at 60 °C, followed by 12 h at room temperature) followed by reduction with ascorbic acid. Iron concentration was determined spectrophotometrically (Tecan Infinite 200 PRO M Plex) against a standard curve of FeCl_3_·6H_2_O (Sigma Aldrich, MilliporeSigma, Burlington, MA, USA) and normalized to total cellular protein content measured by the BCA assay [32].

### 2.6. Statistical Analysis

Data are presented as mean ± standard deviation (SD). Normality was assessed using the Shapiro–Wilk test. Statistical significance between groups was determined using Student’s *t*-test (*p* < 0.05 considered significant). All analyses were performed using GraphPad Prism 8 software (GraphPad Software, San Diego, CA, USA).

## 3. Results

### 3.1. Synthesis of Nanostructured Iron(III) Oxyhydroxides and Their Elemental Composition

A series of 21 samples was prepared by varying the type of humic ligand, precipitation process, and fractionation conditions (Table 1). The obtained preparations were characterized with respect to their total iron and organic carbon content, as well as solubility (S, g/L). Sample designations and relevant synthetic conditions are summarized in Table 2.

Elemental analysis revealed that the content of iron in the Fe(III)–humic preparations was strongly dependent on the type of humic ligand and precipitation conditions. The FA samples showed the highest content of iron (up to 33.5%) and yield (up to 85%), which was attributed to the high solubility and abundance of oxygen-rich functionalities of fulvic acids. Intermediate Fe content (5.7–17.7%) and yields (6–59%) were observed for CHP-based preparations, with the parallel precipitation proving more favorable than the sequential. Whilethe the unsalted control sample (CHPFe) exhibited the highest Fe yield (96%), it also showed the lowest solubility (56.6 g/L), while alcohol precipitated samples reached 120–146 g/L, highlighting the importance of alcohol precipitation for bioavailability. CHS-based products had the lowest Fe incorporation (≤9.6%, ≤54%), which was attributed tooxidative degradation of humic acids. Overall, FA and CHP (Humintech) were most effective for stabilizing Fe(III), whereas CHS was less suitable.

### 3.2. Characterization of Fe(III) Oxyhydroxide Nanoparticles Stabilized with Humic Substances

**Figure 1:**
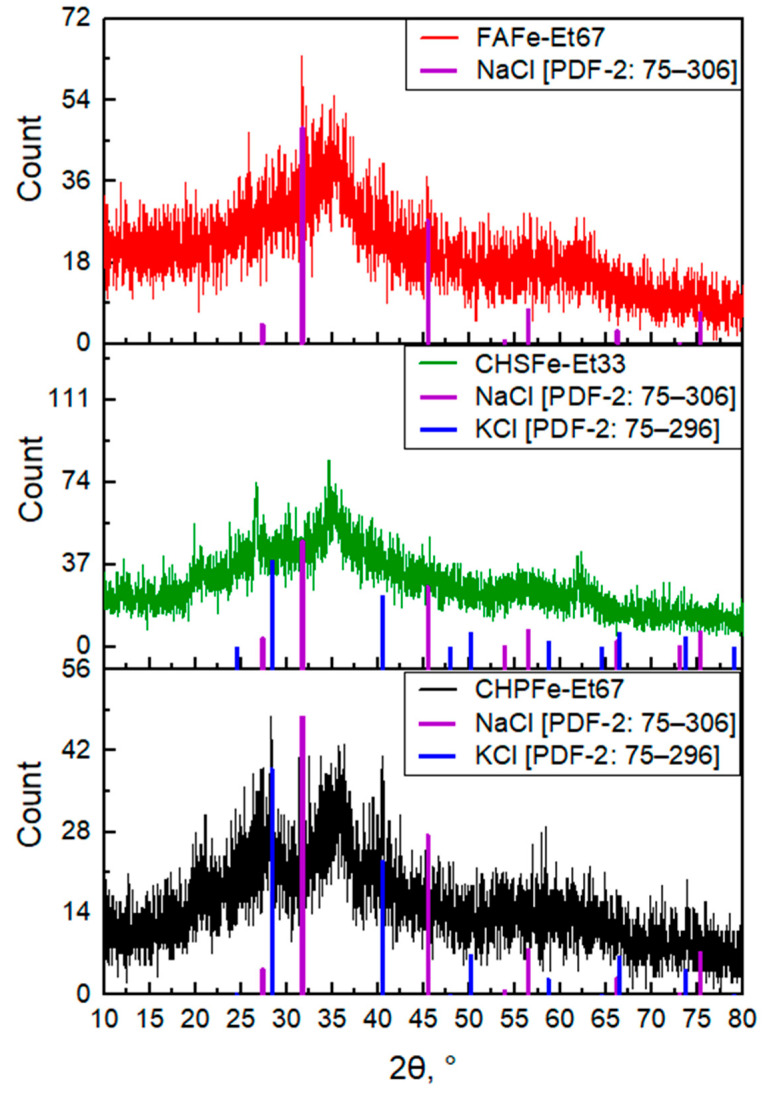
Diffractograms of the studied HS-Fe(III) samples FAFe-Et67; CHSFe-Et33; CHPFe-Et67.

The synthesized samples were characterized by X-ray diffraction and transmission electron microscopy. The XRD patterns of Fe(III) oxyhydroxide nanoparticles stabilized by HS are shown in Figure 1.

Analysis of the obtained diffractograms, revealed that in all samples synthesized using FA and CHS, the main iron-containing phase corresponds to ferrihydrite. This is evidenced by the presence of a characteristic broad amorphous maximum in the range of 2θ ≈ 35°, 63° as well as by comparison with the reference diffraction pattern of ferrihydrite [33]. A similar structure is typical for poorly ordered nanocrystalline phases.

In contrast, CHP samples contained a transition to a more crystalline state—goethite (α–FeOOH)—as evidenced by the appearance of sharp peaks at 2θ = 21°, 33°, 36°, 53°, and 62°, consistent with reference data [34]. This phase transformation is likely attributable to altered synthesis conditions, i.e., prolonged aging of the solution prior to precipitation and low-temperature storage. The literature reports indicate that ferrihydrite can transform into goethite under neutral to slightly alkaline conditions, especially during extended storage in aqueous media at either elevated temperatures or +4 °C [35,36].

The phase composition of the synthesized Fe(III)–HS preparations thus depends on the nature of the stabilizing ligand and synthesis parameters: FA and CHS favor the formation of ferrihydrite, while CHP promotes the crystallization of goethite. The impact of these structural differences on the functional properties of the preparations is of particular interest.

**Figure 2:**
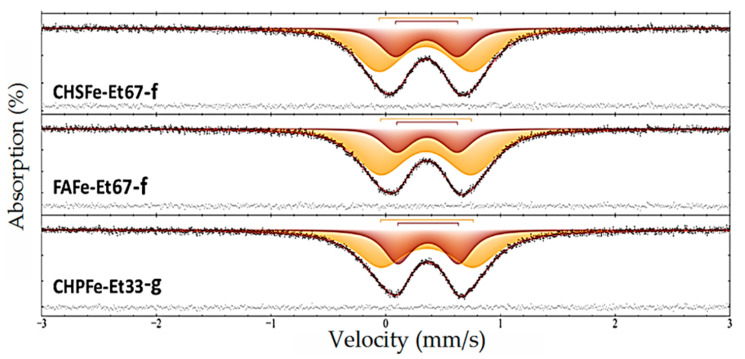
The Mossbauer spectrum of Fe(III)-HS samples at a temperature of 296 K: CHSFe-Et67-f; FAFe-Et-67-f; CHPFe-Et33-g (subspectra simulating experimental data are shown in different colors and the spectrum of residues is shown at the bottom).

Mössbauer spectroscopy confirmed that iron in all the studied samples is present only in the trivalent state and is in an octahedral coordination. The spectra are characterized by a paramagnetic doublet (Figure 2), with isomer shifts (δ ≈ 0.30–0.48 mm/s) and quadrupole splittings (Δ ≈ 0.5–0.8 mm/s) typical for Fe^3+^ in an octahedral ligand field.

These findings demonstrate that regardless of the nature of the macroligand, precipitation process, or solvent composition, iron is always in the same oxidation state and coordination geometry. Depending on the synthesis conditions, iron(III) is stabilized as ferrihydrite or goethite nanophases.

**Figure 3:**
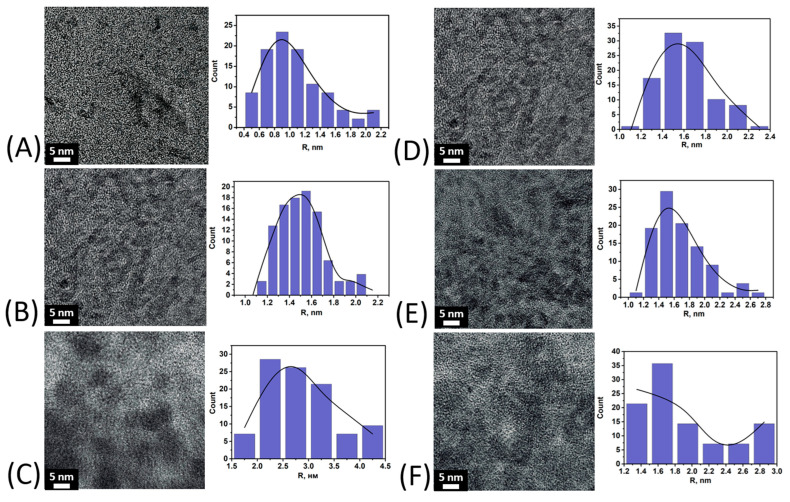
TEM Micrographs of nanoparticles (**left**) and histograms of the distribution of their radii (**right**) for samples: (**A**)—CHPFe-20-I; (**B**)—CHPFe-33-I; (**C**)—CHPFe-43-I; (**D**)—CHPFe-20-II; (**E**)—CHPFe-33-II; (**F**)—CHPFe-43-II.

The morphology and particle size of iron oxyhydroxides in the composition of the obtained preparations were evaluated using TEM. Figure 3 shows micrographs of samples of the CHPFe series obtained by alcohol salting-out. For each sample, in addition to visual evaluation, histograms of the distribution of particle radii were constructed based on the analysis of micrographs.

The micrographs (Figure 3) show discrete nanoparticles of spherical morphology characteristic of all the samples studied. For samples obtained by the method of parallel precipitation with alcohol, an increase in the average radius is observed with an increase in the alcohol content in the system. For CHPFe-20-I, the particle radius is mainly 0.8–1.0 nm, for CHPFe-33-I it is in the range of 1.4–1.6 nm, and for CHPFe-43-I it is 2.0–3.0 nm. The particle size distribution is close to normal. This indicates the formation of relatively homogeneous nanostructures. In a series of samples obtained by sequential alcohol desalination (CHPFe-20-II, CHPFe-33-II, CHPFe-43-II), similar particle radii are observed, mainly in the range of 1.4–1.8 nm. The particles retain a spherical morphology.

**Figure 4:**
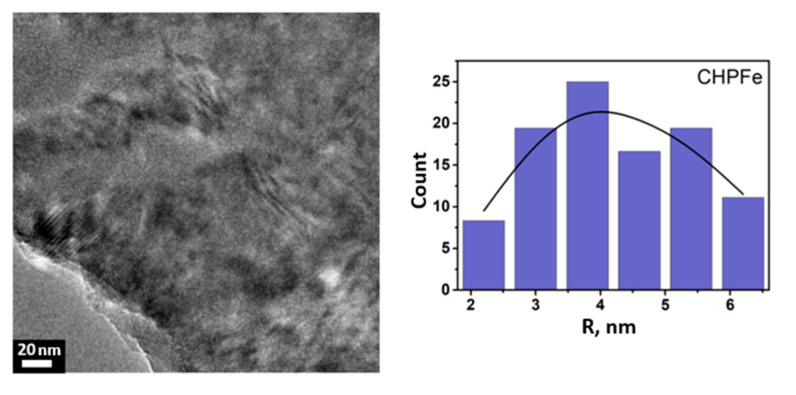
Micrographs of nanoparticles (**left**) and histograms of the nanoparticle size distribution (**right**) in the control sample of CHPFe.

The average radius of the particles obtained by both parallel and sequential alcohol precipitation is in the range of 1–2 nm, which indicates the formation of compact iron(III) nanocomposites stabilized by humic macroligands, regardless of the fractionation method. Considering that the particle size plays a key role in the bioavailability of iron: with decreasing size, their specific surface area and solubility increase, which contributes to more efficient release and subsequent absorption of iron in the body [37,38,39,40]. A control sample of CHPFe was included in the study, which was not subjected to alcohol precipitation. The data is shown in Figure 4.

The TEM data revealed that the CHPFe control sample differed by the presence of large particles from 2 to 6 nm. The largest fraction of particles is concentrated in the 4 nm range. This preparation is characterized by a wide polydispersity and indicates the presence of particles of various sizes in the system.

In summary, the CHP–Fe(III) preparations obtained by gradient alcohol precipitation were characterized by the presence of homogeneous spherical nanoparticles with an average radius of 1–2 nm. In contrast, the CHPFe control sample (without salting-out) contains larger and more polydisperse particles (2–6 nm). Thus, the use of alcohol precipitation enables the isolation of smaller and more homogeneous nanostructures of improved bioavailability.

### 3.3. Cytotoxicity Assessment of Fe(III) Oxyhydroxide Nanoparticles Stabilized with Humic Substances

**Figure 5:**
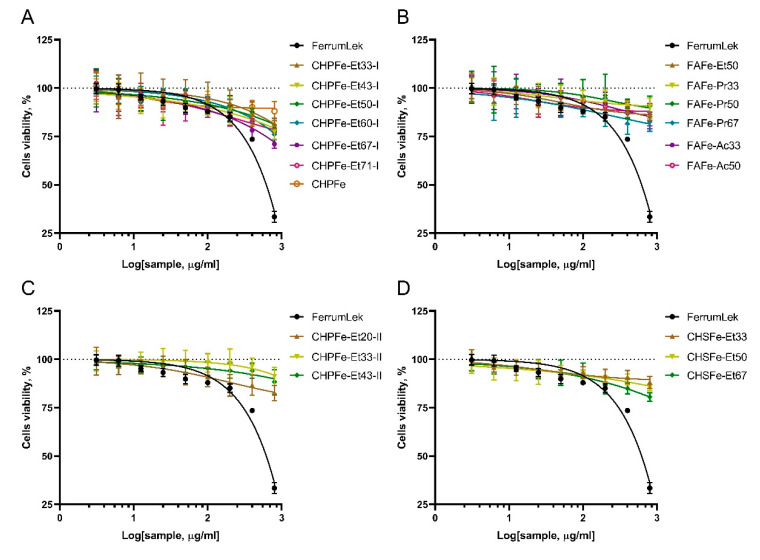
Cytotoxicity profiles of Fe(III)-HS complexes stabilized by Powhumus humic acids with parallel precipitation (**A**), FulvagRa fulvic acids (**B**), Powhumus humic acids with sequential precipitation (**C**) and Sakhalin humates humic acids (**D**) in Caco-2 cells after 24 h exposure. Data represent mean ± SD (*n = 6*). The black solid line represents the dependence of cell viability on the concentration of the reference drug Ferrum Lek^®^. The dashed line indicates the 100% cell viability level.

To establish the preliminary biosafety profile of Fe(III)-HS samples and safe concentration ranges for subsequent iron uptake studies in Caco-2 intestinal epithelial cells, we evaluated the cytotoxicity of Fe(III)-HS complexes and the reference compound Ferrum Lek® using the Neutral Red Uptake assay. Cells were exposed to concentrations spanning 3.125–800 μg/mL for 24 h (Figure 5 and Figure S1).

All Fe(III)-HS samples exhibited remarkably low cytotoxicity across the tested range. Viability remained >80% at concentrations ≤ 200 μg/mL for most complexes. Even at the highest concentrations (400–800 μg/mL), cytotoxicity was minimal. Average viability at 800 μg/mL was 85.3% ± 4.1% for FulvagRa-based complexes (e.g., FAFe-Pr50: 98.7% ± 1.2%). Powhumus sequential-precipitation samples (e.g., CHPFe-Et33-II) showed no significant viability reduction (101.9% ± 3.4% at 800 μg/mL) at all. Only Sakhalin humates (e.g., CHSFe-Et67) demonstrated moderate reduction (79.0% ± 2.1% at 800 μg/mL).

Critically, IC50 values were unattainable for all Fe(III)-HS complexes, as viability never fell below 50%, confirming exceptional biocompatibility.

This negligible cytotoxicity starkly contrasted with the performance of the reference drug Ferrum Lek^®^. As shown in Figure 5, Ferrum Lek^®^ exhibited pronounced toxicity, with viability plummeting to 35.7% ± 2.8% at 800 μg/mL. Statistical analysis confirmed significant cell death at concentrations > 200 μg/mL (*p* < 0.001 vs. untreated controls; S1), yielding an IC50 of 412 μg/mL. This aligns with clinical reports of gastrointestinal adverse effects associated with conventional iron supplements and underscores a critical advantage of humic stabilization.

The minimal cytotoxicity of Fe(III)-HS complexes likely stems from the intrinsic properties of humic substances. Their known antioxidant capacity and membrane-stabilizing effects appear to mitigate iron-induced oxidative stress—a common toxicity mechanism of traditional iron supplements. This protective effect was particularly evident in FAFe-Pr50 and CHPFe-Et33-II, which maintained near-baseline viability even at maximal concentrations.

Based on these findings, concentration 50 μg/mL was selected for subsequent iron uptake studies, as all Fe(III)-HS complexes maintained >90% cell viability within this range.

### 3.4. Fe(III) Oxyhydroxide Nanoparticles Stabilized with Humic Substances Iron Bioavailability Assessment

**Figure 6:**
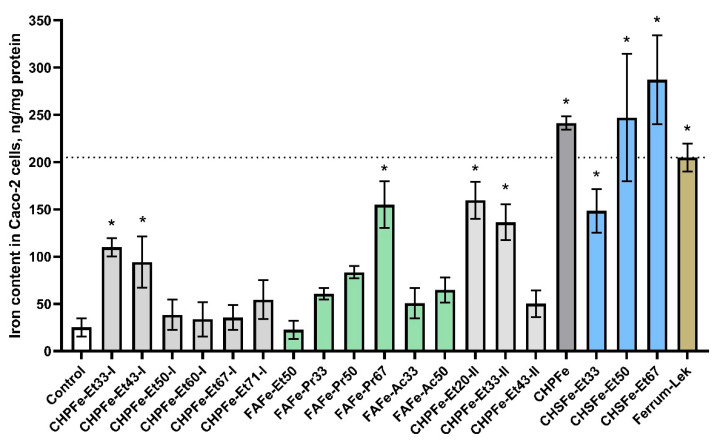
Comparative intracellular iron accumulation in Caco-2 cells after 24 h exposure to humic-stabilized nanoferrotherapeutics and Ferrum Lek^®^ (50 μg Fe/mL). Data presented as mean ± SD. * *p* < 0.05 vs. untreated control. The color of the columns indicates the clustering of the samples (with the control and reference groups), based on the type of macroligand and solvent used for synthesis (see Table 2). The dashed line corresponds to the intracellular iron level in cells incubated with the reference drug Ferrum Lek^®^.

To evaluate the functional efficacy of synthesized Fe(III)-HS complexes, we quantified intracellular iron accumulation in Caco-2 cells following 24 h exposure at a standardized iron concentration of 50 μg/mL. The results, presented in Table 3 and Figure 6 reveal striking differences in absorption efficiency across sample types, with fold-change values relative to untreated controls in Table 3 providing critical insights into structure-activity relationships.

The reference drug Ferrum Lek^®^ demonstrated significant iron delivery capability, increasing intracellular iron levels by 10.4-fold compared to untreated controls (204.9 ± 14.6 vs. 25.1 ± 9.6 ng/mg protein). However, this performance was substantially surpassed by several humic-stabilized Fe(III)-HS. Most notably, CHSFe-Et67 achieved unprecedented iron accumulation (287.2 ± 47.0 ng/mg protein; 14.6-fold increase), while CHSFe-Et50 (247.1 ± 67.6 ng/mg protein; 12.5-fold) and surprisingly the unsalted CHPFe control (241.3 ± 6.9 ng/mg protein; 12.2-fold) also exceeded Ferrum Lek^®^ by >20%.

A clear hierarchy of performance emerged when evaluating formulation parameters. Sequential precipitation generally enhanced bioavailability versus parallel methods, with CHPFe-Et20-II (8.1-fold) and CHPFe-Et33-II (6.9-fold) outperforming their parallel-precipitated counterparts. Solvent polarity significantly influenced absorption, where higher alcohol concentrations correlated with improved delivery—particularly evident in the FAFe-Pr series where increasing isopropanol from 33% to 67% boosted uptake from 3.1-fold to 7.9-fold. This trend suggests that alcohol precipitation optimizes the lipophilicity-hydrophilicity balance critical for membrane permeation, aligning with our earlier hypothesis about molecular transport optimization.

The Sakhalin humate (CHS) series exhibited exceptional performance, with all formulations surpassing Ferrum Lek^®^. The progressive enhancement from CHSFe-Et33 (7.5-fold) to CHSFe-Et67 (14.6-fold) indicates that higher ethanol concentrations during precipitation progressively refine the nanostructure for optimal absorption. This contrasts with Powhumus-derived samples where sequential precipitation (CHPFe-Et20-II, 8.1-fold) outperformed parallel methods but remained below Sakhalin benchmarks. The fulvic acid (FA) series showed more variable results, with FAFe-Pr67 (7.9-fold) approaching Ferrum Lek^®^ efficacy while FAFe-Et50 (1.1-fold) showed negligible uptake—highlighting how solvent selection critically determines functional outcomes.

## 4. Discussion

This study demonstrates the possibility of synthesizing water-soluble iron(III) oxyhydroxide nanoparticles stabilized with humic macroligands using alcohol precipitation methods. A series of 21 samples of nanostructured HS-Fe(III) complexes were obtained by varying the type of ligand (CHP, CHS, FA), solvent (ethanol, isopropanol, acetone), and precipitation method. The type of humic ligand and precipitation conditions had a significant effect on the physicochemical properties of the nanocomposites. The samples based on fulvate ligands showed the highest iron content (up to 33.5%) and solubility, whereas the humate-based samples had lower values. Precipitation methods yielded more homogeneous and soluble products compared to sequential fractionation methods. X-ray diffraction and Mössbauer spectroscopy showed the formation of a low-crystalline ferrihydrite or goethite phase depending on the synthetic conditions. Ferrihydrite was obtained in the preparations, which were immediately precipitated with alcohol and dried, while goethite was obtained in the preparations which were precipitated after 12 h exposure after synthesis. TEM results showed that alcohol precipitation allowed for preparation of homogeneous spherical nanoparticles with a radius of 1–2 nm, whereas the control sample, CHPFe, had larger and polydisperse particles ranging from 2 to 6 nm.

Our results demonstrate that the presence of HS macroligands confers exceptional cytoprotection: all Fe(III)-HS formulations maintained >80% cell viability even at 800 μg/mL, with IC50 values unattainable due to negligible toxicity. This contrasts greatly with the data for Ferrum Lek^®^ (IC_50_ = 412 μg/mL) and is in agreement with clinical reports of conventional iron toxicity. The antioxidant properties of HS [11] might mitigate iron-induced oxidative damage—a key toxicity mechanism of traditional supplements.

The obtained Fe(III)-HS nanocomposites possessed essential bioavailability [41,42,43]. The most bioavailable CHP fraction was precipitated at low concentrations of ethanol (Et20–33%). Under these conditions, the more hydrophilic areas in the otherwise hydrophobic leonardite-derived humic acids are preferentially precipitated to produce nanoparticles with optimal solubility and transport properties. In contrast, the most bioavailable preparations with more hydrophilic FA ligands were obtained at high concentrations of alcohol (Pr 67%), which favor precipitation of the most hydrophobic molecular fractions. This suggests that effective membrane permeability is brought about by a delicate hydrophilic–lipophilic balance, which can be modulated by solvent-driven fractionation.

The best results were demonstrated for the CHSFe-Et67 sample, reaching an intracellular iron level of 287.2 ng Fe/mg protein, which is 40% higher than that of Ferrum Lek^®^, despite the relatively low iron content (5.8–9.6%). The 14.6-fold iron uptake observed for CHSFe-Et67 demonstrates how nanostructuring of iron oxyhydroxide with HS overcomes absorption barriers inherent to conventional therapies. Future studies should explore in vivo correlation and molecular transport mechanisms.

The obtained results enable us to conclude that HS provide a suitable platform for iron delivery, leveraging their natural polyfunctionality to form stable nanocomplexes while exhibiting inherent biocompatibility [9,10,11,12,13].

## Figures and Tables

**Table 1:** Parameters of sequential and parallel precipitation of HS-Fe fractions.

Solvent Content in the Mixture, %	Parallel Precipitation		Sequential Precipitation	
	Volume of Fe(III)-HS Mixture, mL	Solvent Volume, mL	Volume of Fe(III)-HS Mixture, mL	Solvent Volume, mL
20	50	12.5	100	25
33	50	25		25
43	50	37.5		25
50	50	50		25
60	50	75		50
67	50	100		50
71	50	125		50

**Table 2:** Description and characteristics of the Fe(III)-HS samples synthesized in this study and characteristics.

Name *	Fe Content, %	Fe Yield, %	C Content, %	C Yield, %	S, g/L
CHPFe	25.1	96	17.9	32	56.6
CHPFe-Et20-I	16.2	27	25.8	19	144.5
CHPFe-Et33-I	13.1	56	31.2	63	125.9
CHPFe-Et43-I	11.7	56	29.5	62	141.8
CHPFe-Et50-I	11.6	50	32.6	69	132.6
CHPFe-Et60-I	12.6	57	32.6	74	139.7
CHPFe-Et67-I	12.2	58	32.1	82	140.7
CHPFe-Et71-I	11.9	59	32.7	74	133.8
CHPFe-Et20-II	8.3	17	34.3	23	143.3
CHPFe-Et33-II	17.7	35	23.7	26	120.1
CHPFe-Et43-II	5.7	6	38.6	15	145.7
FAFe-Et50	27.2	48	16.9	49	-
FAFe-Et67	15.6	43	21.5	31	-
FAFe-Pr33	33.5	72	15.9	22	-
FAFe-Pr50	33.5	83	21.2	53	-
FAFe-Pr67	12.7	77	28.0	57	-
FAFe-Ac33	30.3	85	16.1	28	-
FAFe-Ac50	23.4	85	20.6	47	-
CHSFe-Et33	9.3	43	-	-	-
CHSFe-Et50	9.6	54	-	-	-
CHSFe-Et67	5.8	54	-	-	-

* CHP, CHS, and FA stands for the type of macroligand used; Et, Pr, and Ac stands for the percentage of solvent (ethanol, isopropanol, and acetone, respectively); I and II stands for the parallel and sequential precipitation, respectively, the samples without extensions I and II are prepared by parallel precipitation except for CHPFe, which was used as a control and was obtained without solvent precipitation.

**Table 3:** Iron content (ng/mg protein) in Caco-2 cells after 24 h incubation with Fe(III)-HS complexes (M ± SD).

Sample	Iron Content (ng/mg Protein)	Fold-Change vs. Control
Control	25.1 ± 9.6	1.0
CHPFe-Et33-I	109.9 ± 9.8	5.6
CHPFe-Et43-I	94.3 ± 27.2	4.8
CHPFe-Et50-I	38.4 ± 16.0	2.0
CHPFe-Et60-I	33.7 ± 18.0	1.7
CHPFe-Et67-I	35.5 ± 13.1	1.8
CHPFe-Et71-I	54.4 ± 20.6	2.8
FAFe-Et50	22.6 ± 9.6	1.1
FAFe-Pr33	60.7 ± 6.1	3.1
FAFe-Pr50	83.5 ± 6.7	4.2
FAFe-Pr67	154.8 ± 24.7	7.9
FAFe-Ac33	50.7 ± 16.0	2.6
FAFe-Ac50	64.8 ± 13.2	4.8
CHPFe-Et20-II	159.7 ± 19.7	8.1
CHPFe-Et33-II	136.4 ± 19.0	6.9
CHPFe-Et43-II	50.2 ± 14.0	2.5
CHPFe	241.3 ± 6.9	12.2
CHSFe-Et33	148.6 ± 23.1	7.5
CHSFe-Et50	247.1 ± 67.6	12.5
CHSFe-Et67	287.2 ± 47.0	14.6
Ferrum-Lek	204.9 ± 14.6	10.4

## Data Availability

Original contributions presented in this study are included in the article. Further inquiries can be directed to the corresponding authors.

## Supplementary Materials

The following supporting information can be downloaded at: https://www.mdpi.com/article/10.3390/nano15181400/s1, Supplementary File contains the individual cytotoxicity data of the samples towards Caco-2 cells.
