# Supplementary material for: Humics-Functionalized Iron(III) Oxyhydroxides as Promising Nanoferrotherapeutics: Synthesis, Characterization, and Efficacy in Iron Delivery
Source: Nanomaterials (Basel). 2025 Sep 11;15(18):1400. doi: 10.3390/nano15181400 (PMC12472974; doi:10.3390/nano15181400)
Supplement: Supplementary file 1 [file nanomaterials-15-01400-s001.zip › nanomaterials-3829190-supplementary.pdf]

nanomaterials

MDPI

Article

Humics-Functionalized Iron(III) Oxyhydroxides as Promising Nanoferrotherapeutics: Synthesis, Characterization, and Efficacy in Iron Delivery

Anastasiya M. Zhirkova [1,*], Maria V. Zykova [2,*], Evgeny E. Buyko [2], Karina A. Ushakova [1], Vladimir V. Ivanov [2], Denis A. Pankratov [1], Elena V. Udut [2], Lyudmila A. Azarkina [2], Sergey R. Bashirov [2], Evgenii V. Plotnikov [3], Alexey N. Pestryakov [3], Mikhail V. Belousov [2] and Irina V. Perminova [1]

[1] Department of Chemistry, Lomonosov Moscow State University, Leninskiye Gory 1-3, Moscow 119991, Russia; kiorika@bk.ru (K.A.U.); pankratov@radio.chem.msu.ru (D.A.P.); iperminova@gmail.com (I.V.P.)
[2] Pharmaceutical Faculty, Siberian State Medical University, Tomsk 634050, Russia; buykoevgen@yandex.ru (E.E.B.); ivanovvv1953@gmail.com (V.V.I.); evu8@mail.ru (E.V.U.); ludmila_logvinova@mail.ru (L.A.A.); bars-tomsk@rambler.ru (S.R.B.); mvb63@mail.ru (M.V.B.)
[3] Research School of Chemistry and Applied Biomedical Sciences, Tomsk Polytechnic University, Tomsk 634050, Russia; plotnikov.e@mail.ru (E.V.P.); pestryakov@tpu.ru (A.N.P.)
* Correspondence: anastasiia.zhirkova@chemistry.msu.ru (A.M.Z.); huminolog@mail.ru (M.V.Z.); Tel.: +7-9104951397 (A.M.Z.); +7-3822901101 (ext. 1918) (M.V.Z.)

Cytotoxicity assessment of Fe(III) oxyhydroxide nanoparticles stabilized with humic substances

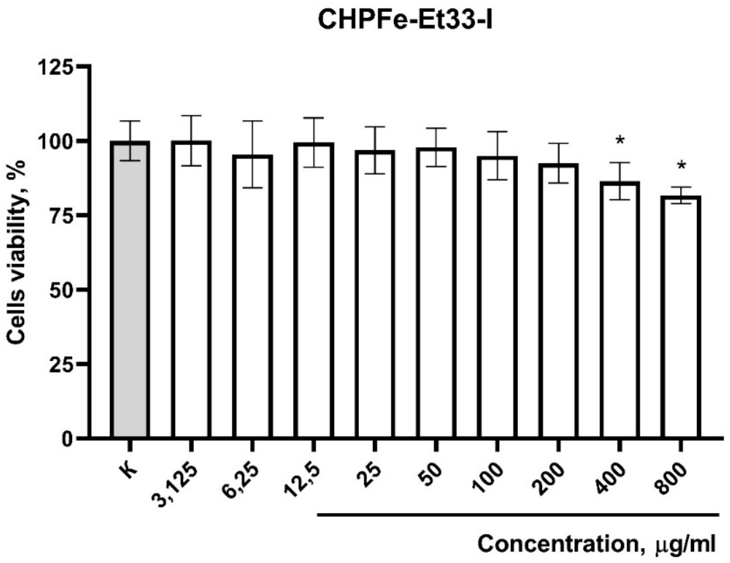

Figure S1. Cytotoxicity profile of CHPFe-Et33-I Fe(III) oxyhydroxide nanoparticles stabilized with humic substances in Caco-2 cells after 24 h exposure. Data represent mean ± SD (n=6). *$p < 0.05$ vs. untreated control.

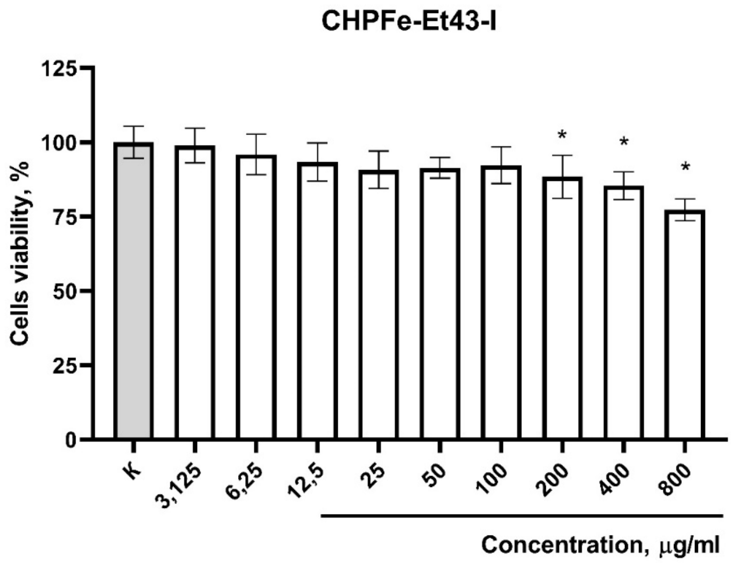

Figure S2. Cytotoxicity profile of CHPFe-Et43-I Fe(III) oxyhydroxide nanoparticles stabilized with humic substances in Caco-2 cells after 24 h exposure. Data represent mean ± SD (n=6). *$p < 0.05$ vs. untreated control.

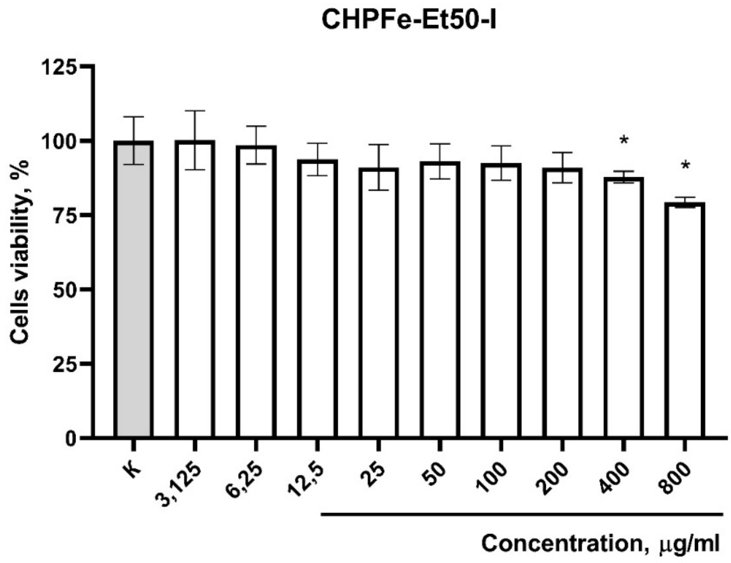

Figure S3. Cytotoxicity profile of CHPFe-Et50-I Fe(III) oxyhydroxide nanoparticles stabilized with humic substances in Caco-2 cells after 24 h exposure. Data represent mean ± SD (n=6). *$p < 0.05$ vs. untreated control.

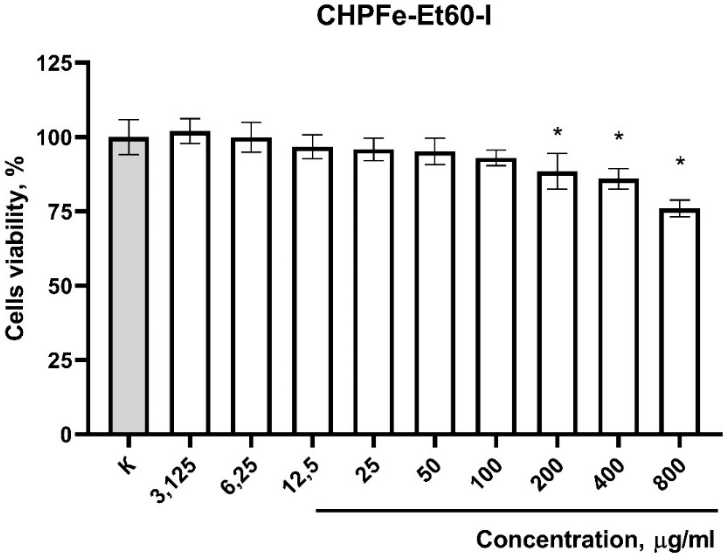

Figure S4. Cytotoxicity profile of CHPFe-Et60-I Fe(III) oxyhydroxide nanoparticles stabilized with humic substances in Caco-2 cells after 24 h exposure. Data represent mean ± SD (n=6). *$p < 0.05$ vs. untreated control.

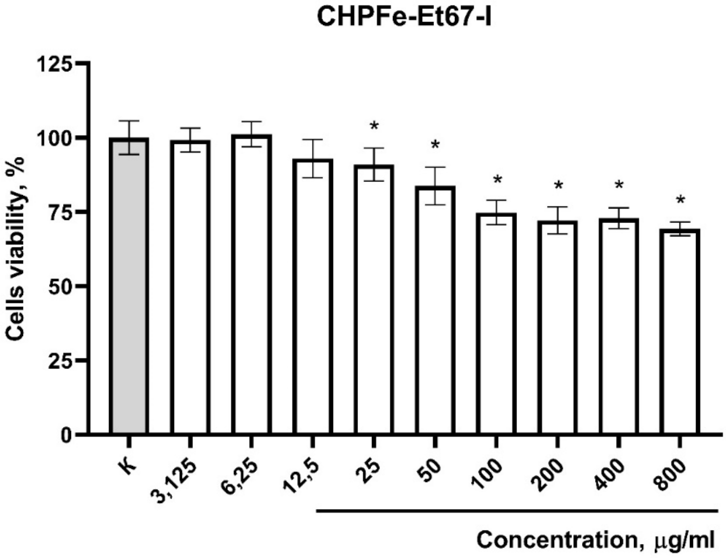

Figure S5. Cytotoxicity profile of CHPFe-Et67-I Fe(III) oxyhydroxide nanoparticles stabilized with humic substances in Caco-2 cells after 24 h exposure. Data represent mean ± SD (n=6). *$p < 0.05$ vs. untreated control.

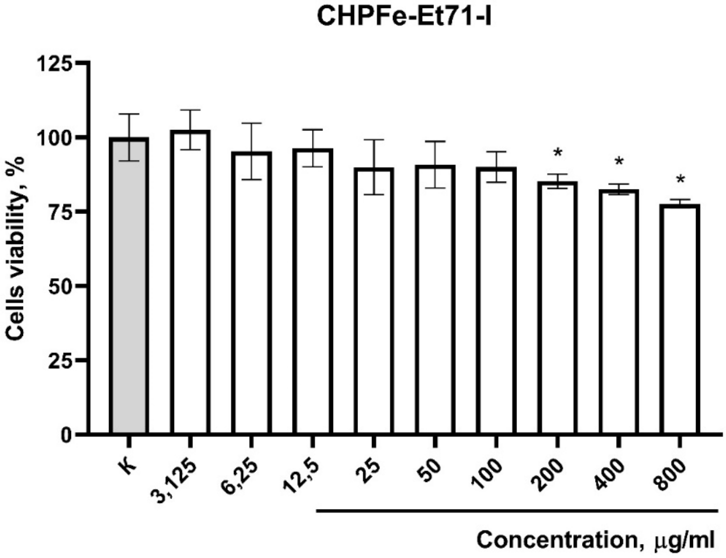

Figure S6. Cytotoxicity profile of CHPFe-Et71-I Fe(III) oxyhydroxide nanoparticles stabilized with humic substances in Caco-2 cells after 24 h exposure. Data represent mean ± SD (n=6). *$p < 0.05$ vs. untreated control.

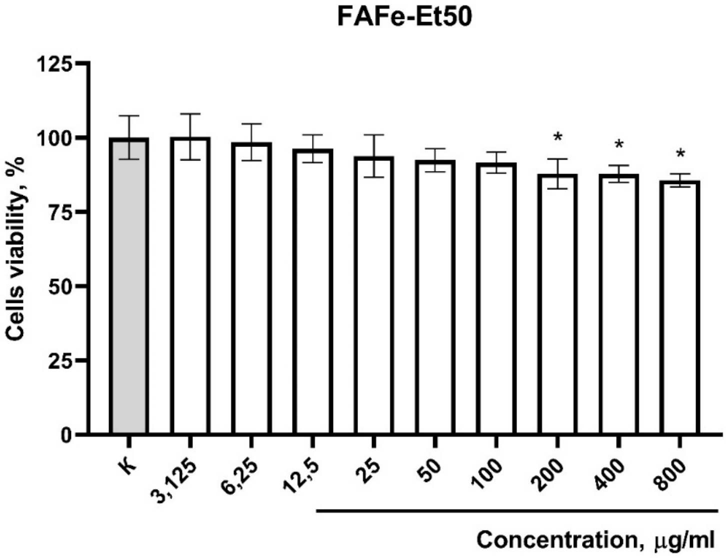

Figure S7. Cytotoxicity profile of FAFe-Et50 Fe(III) oxyhydroxide nanoparticles stabilized with humic substances in Caco-2 cells after 24 h exposure. Data represent mean ± SD (n=6). *$p < 0.05$ vs. untreated control.

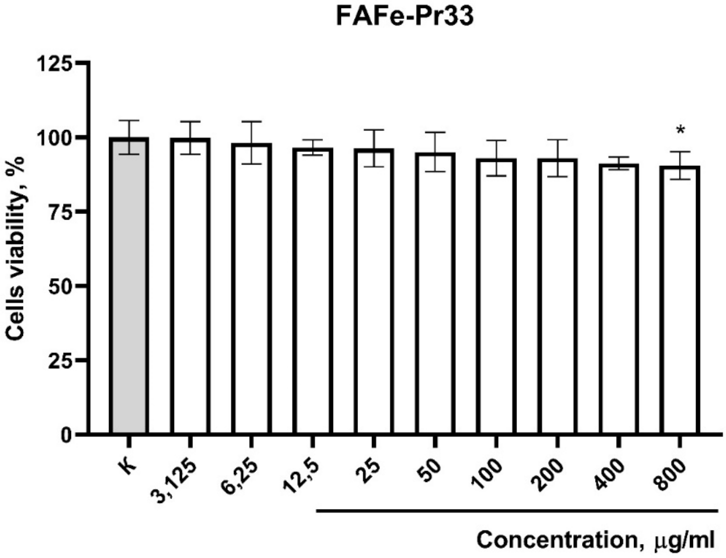

Figure S8. Cytotoxicity profile of FAFe-Pr33 Fe(III) oxyhydroxide nanoparticles stabilized with humic substances in Caco-2 cells after 24 h exposure. Data represent mean ± SD (n=6). *$p < 0.05$ vs. untreated control.

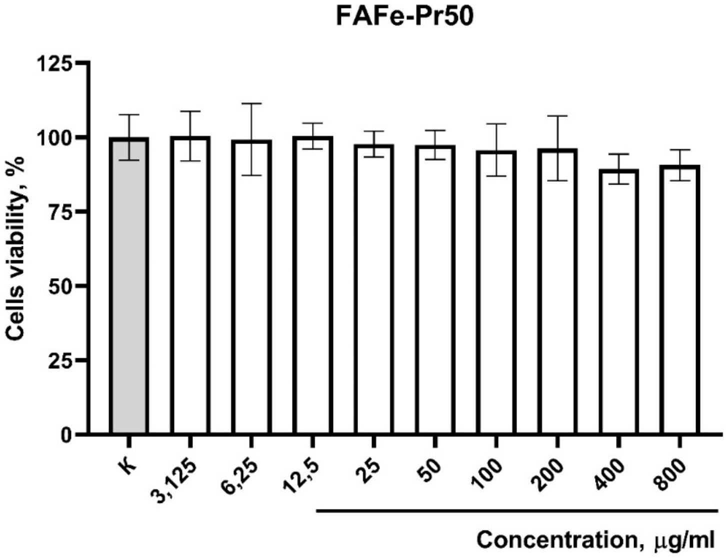

Figure S9. Cytotoxicity profile of FAFe-Pr50 Fe(III) oxyhydroxide nanoparticles stabilized with humic substances in Caco-2 cells after 24 h exposure. Data represent mean ± SD (n=6). *$p < 0.05$ vs. untreated control.

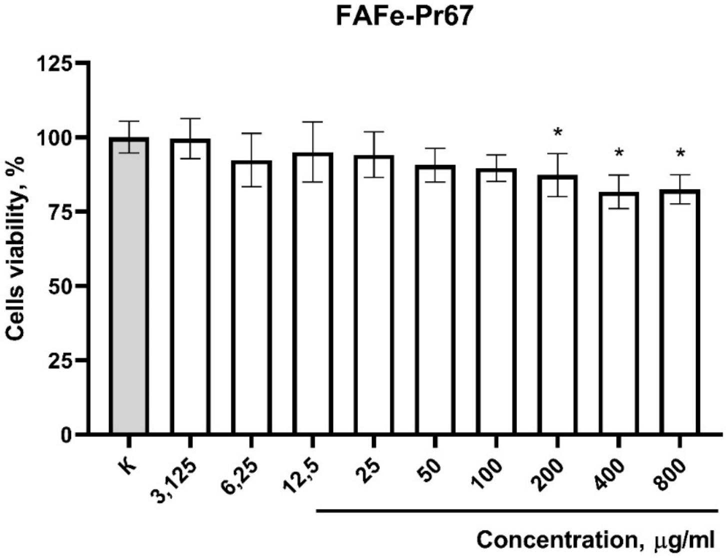

Figure S10. Cytotoxicity profile of FAFe-Pr67 Fe(III) oxyhydroxide nanoparticles stabilized with humic substances in Caco-2 cells after 24 h exposure. Data represent mean ± SD (n=6). *p < 0.05 vs. untreated control.

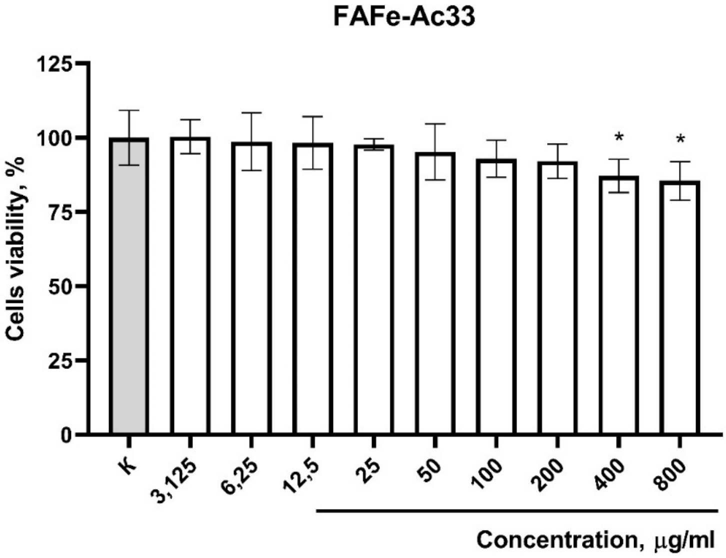

Figure S11. Cytotoxicity profile of FAFe-Ac33 Fe(III) oxyhydroxide nanoparticles stabilized with humic substances in Caco-2 cells after 24 h exposure. Data represent mean ± SD (n=6). *p < 0.05 vs. untreated control.

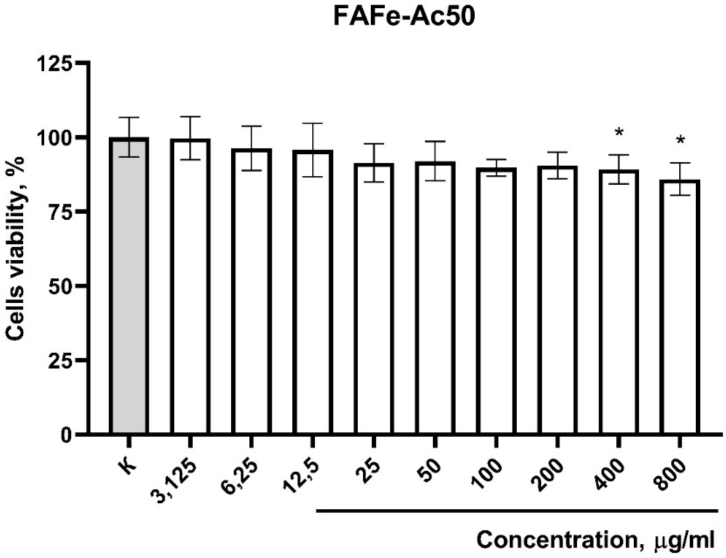

Figure S12. Cytotoxicity profile of FAFe-Ac50 Fe(III) oxyhydroxide nanoparticles stabilized with humic substances in Caco-2 cells after 24 h exposure. Data represent mean ± SD (n=6). *p < 0.05 vs. untreated control.

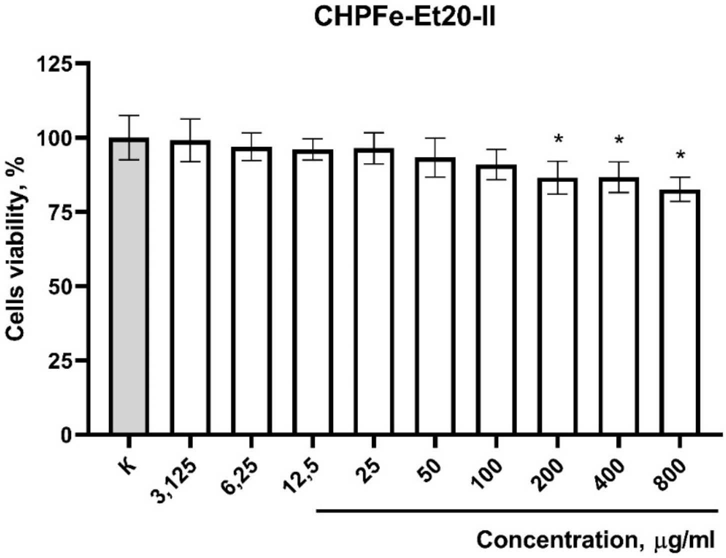

Figure S13. Cytotoxicity profile of CHPFe-Et20-II Fe(III) oxyhydroxide nanoparticles stabilized with humic substances in Caco-2 cells after 24 h exposure. Data represent mean ± SD (n=6). *p < 0.05 vs. untreated control.

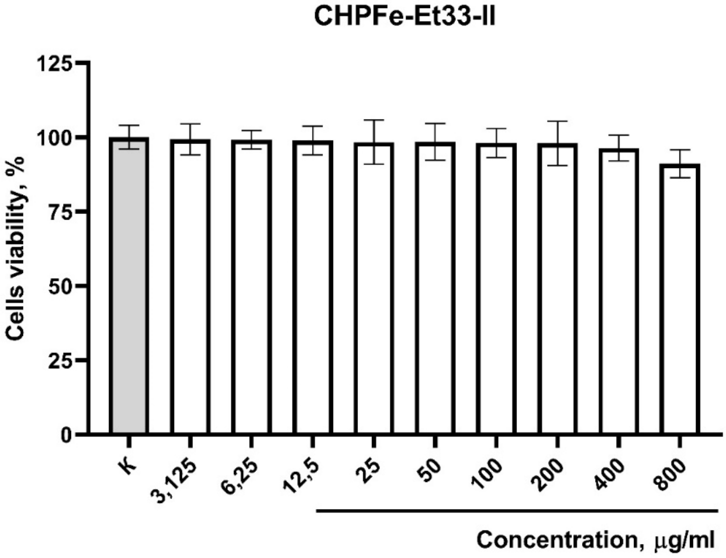

Figure S14. Cytotoxicity profile of CHPFe-Et33-II Fe(III) oxyhydroxide nanoparticles stabilized with humic substances in Caco-2 cells after 24 h exposure. Data represent mean ± SD (n=6). *$p < 0.05$ vs. untreated control.

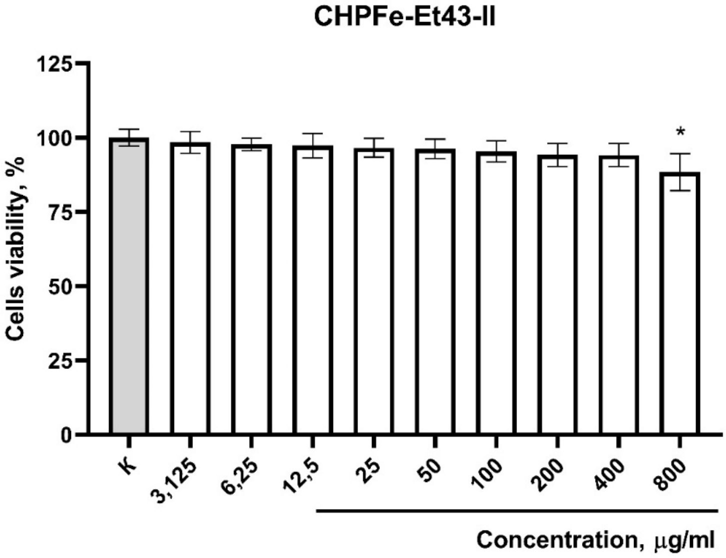

Figure S15. Cytotoxicity profile of CHPFe-Et43-II Fe(III) oxyhydroxide nanoparticles stabilized with humic substances in Caco-2 cells after 24 h exposure. Data represent mean ± SD (n=6). *$p < 0.05$ vs. untreated control.

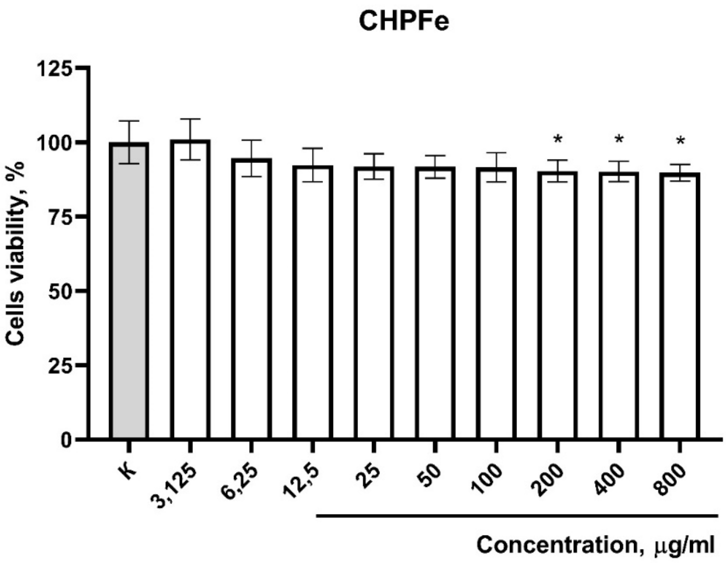

Figure S16. Cytotoxicity profile of initial sample CHPFe Fe(III) oxyhydroxide nanoparticles stabilized with humic substances in Caco-2 cells after 24 h exposure. Data represent mean ± SD (n=6). *$p < 0.05$ vs. untreated control.

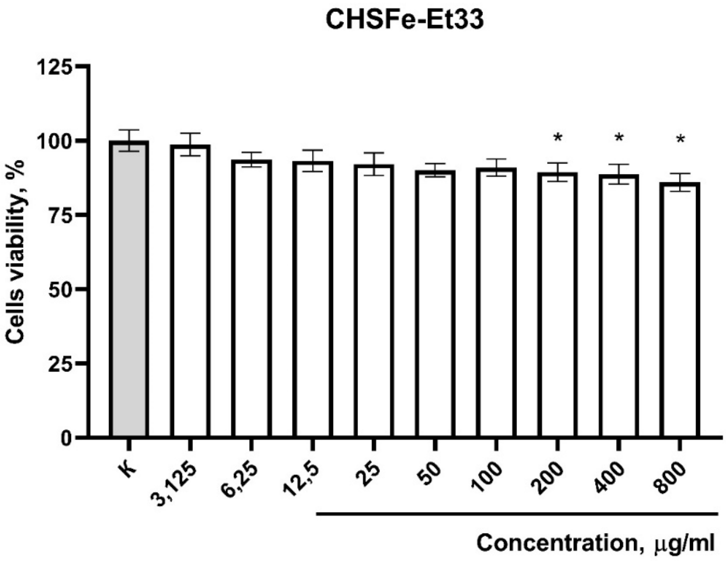

Figure S17. Cytotoxicity profile of CHSFe-Et33 Fe(III) oxyhydroxide nanoparticles stabilized with humic substances in Caco-2 cells after 24 h exposure. Data represent mean ± SD (n=6). *$p < 0.05$ vs. untreated control.

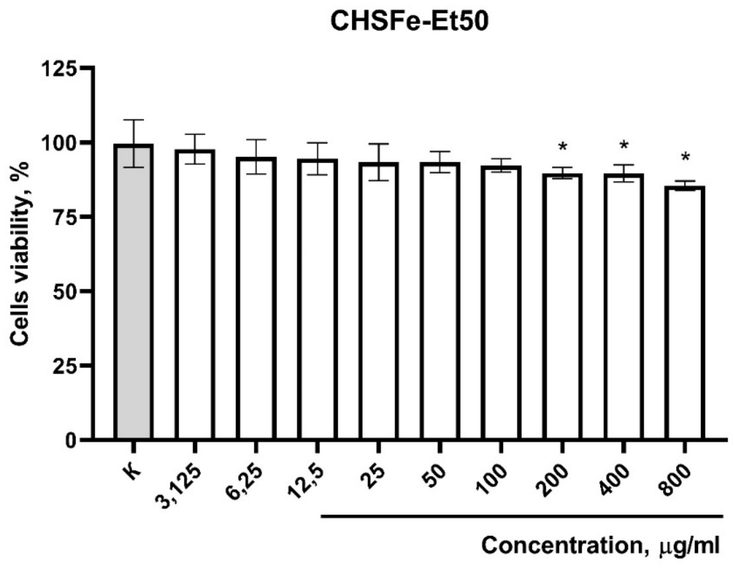

Figure S18. Cytotoxicity profile of CHSFe-Et50 Fe(III) oxyhydroxide nanoparticles stabilized with humic substances in Caco-2 cells after 24 h exposure. Data represent mean ± SD (n=6). *$p < 0.05$ vs. untreated control.

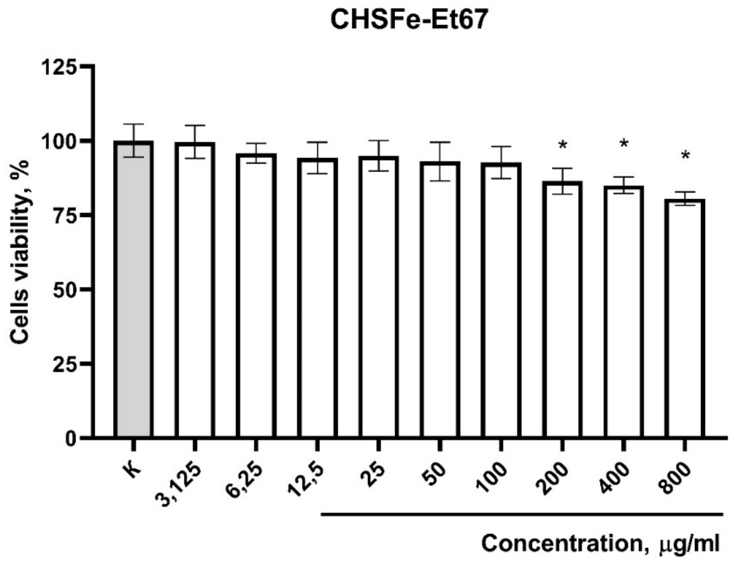

Figure S19. Cytotoxicity profile of CHSFe-Et67 Fe(III) oxyhydroxide nanoparticles stabilized with humic substances in Caco-2 cells after 24 h exposure. Data represent mean ± SD (n=6). *$p < 0.05$ vs. untreated control.

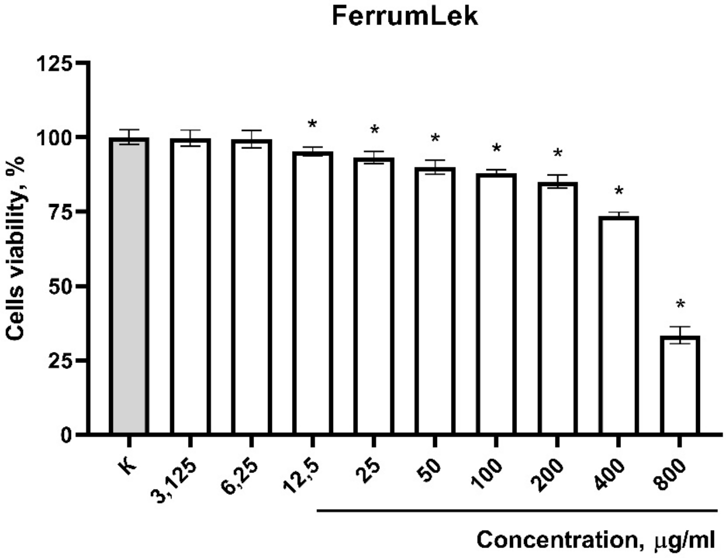

Figure S20. Cytotoxicity profile of reference drug FerrumLek in Caco-2 cells after 24 h exposure. Data represent mean ± SD (n=6). *$p < 0.05$ vs. untreated control.
